# Recalcitrant Neuropathies in the Upper Extremity

**DOI:** 10.1016/j.jhsg.2023.03.002

**Published:** 2023-03-28

**Authors:** Hannah C. Langdell, Steven L. Zeng, Tyler S. Pidgeon, Suhail K. Mithani

**Affiliations:** ∗ Division of Plastic, Reconstructive, Maxillofacial and Oral Surgery, Duke University Medical Center, Durham, NC; † Department of Orthopaedic Surgery, Duke University Medical Center, Durham, NC

**Keywords:** Carpal tunnel syndrome, Cubital tunnel syndrome, Hypothenar fat pad flap, Recurrent neuropathies

## Abstract

Carpal and cubital tunnel syndrome can cause debilitating pain and weakness in the hand and upper extremities. Although most patients have a resolution of their symptoms after primary decompression, managing those with recalcitrant neuropathies is challenging. The etiology of persistent, recurrent, or new symptoms is not always clear and requires careful attention to the history and physical examination to confirm the diagnosis or consider other causes prior to committing to surgery. Nevertheless, revision surgery is often needed in the setting of recalcitrant neuropathies in order to improve patients’ symptoms. Revision surgery typically entails wide exposure and neurolysis to release residual compression. In addition, vascularized tissue and nerve wraps have been routinely used to create a favorable perineural environment that decreases recurrent scar formation. This review discusses the etiologies of recalcitrant upper extremity neuropathies, the current treatment options, and surgical outcomes.

Peripheral nerve compression in the upper extremity can lead to predictable patterns of neurological symptoms and functional impairment depending on the site of focal compression. As nerves pass through enclosed or narrow spaces, they can be subjected to increased pressure, leading to microvascular changes within the nerves and myelin sheaths. While peripheral nerves typically can tolerate low pressures, sustained higher pressures can result in changes in permeability, increased interfascicular edema, conductivity impedance, and arterial ischemia. Pressures of 30 mm Hg can lead to increased permeability and edema, while 70 mm Hg can cause complete ischemia.^1^^,^^2^ These changes ultimately cause nerve fibrosis, demyelination, and axonal degeneration in the end stages. Physical examination findings of muscle wasting, electrodiagnostic studies, and nerve conduction studies can help ascertain the timing, severity, and localization of the nerve injury. The degree of injury can, in turn, predict the prognosis of recovery and guide treatment options.

Carpal tunnel syndrome is the most common upper-extremity compression neuropathy, with an annual incidence of 0.1% to 0.35%. Ulnar nerve compression at the elbow, or cubital tunnel syndrome, is the second most common, with an annual incidence of 0.02%.^3^ These compression syndromes have reliable and well-studied conservative and operative management strategies. Overall, carpal tunnel release (CTR) and cubital tunnel decompression successfully relieve patients’ symptoms. However, some patients have persistent, recurrent, or new symptoms that require secondary operations. Approximately 2% to 10% of patients require carpal tunnel revision surgery, while rates of revision surgery for cubital tunnel syndrome are 12% to 25%.^4^^,^^5^ In this review, we discuss the etiology, surgical techniques, and associated outcomes of recalcitrant carpal and cubital tunnel syndromes.

## Recalcitrant Carpal Tunnel Syndrome

### Anatomy and etiology

The median nerve originates from the C6-T1 nerve roots as a convergence of the medial and lateral cords of the brachial plexus. Near the elbow, the median nerve passes under the ligament of Struthers. It travels deep to the lacertus fibrosis and then between the two heads of the pronator teres muscle. The nerve courses deep to the proximal fibrous arch of the flexor digitorum superficialis (FDS) muscle and then between the FDS and flexor digitorum profundus (FDP) muscles in the forearm. The anterior interosseus nerve (AIN) branches off the median nerve 2–8 cm distal to the medial epicondyle either under or distal to the pronator teres muscle and then runs between the FDP and FDS muscles, whereas the median nerve proper travels through the carpal tunnel.^6^

When patients present to the clinic after a CTR with median nerve symptoms, it is important to differentiate between persistent, recurrent, or new symptoms. This distinction will guide the timing and aid in selecting the proper treatment course. Persistent CTS is defined as no period of symptom relief after CTR and is most often the result of the incomplete release of the transverse carpal ligament or antebrachial fascia. Other less common median neuropathies, such as supracondylar process syndrome, pronator syndrome, lacertus syndrome, and AIN syndrome, should also be considered. In addition to numbness, paresthesias, or weakness in the median nerve distribution, patients with supracondylar process syndrome also present with pain exacerbated by forearm extension and pronation, tenderness above the medial epicondyle, vascular compromise presenting as ischemic pain or thrombosis, fracture of the supracondylar process, and a palpable supracondylar process.^7^ Additionally, the supracondylar process can easily be visualized on oblique X-ray views. Pronator syndrome is distinguished from CTS by negative Tinel and Phalen signs over the carpal tunnel and a lack of nocturnal symptoms since wrist flexion should not worsen symptoms.^8^ Furthermore, patients with pronator syndrome may have sensory changes over the proximal thenar eminence since the palmar cutaneous branch originates distal to the site of compression. On physical examination, patients may have a positive Gainor compression test, tenderness over pronator teres 6 cm distal to the elbow crease, or forearm tenderness aggravated by resisted forearm pronation or long finger FDS contraction.^8^^,^^9^ Lacertus syndrome is a type of exertional compartment syndrome often seen in throwing athletes, which presents as achy pain over the medial elbow. The pain develops after activity and resolves with rest.^10^ Finally, AIN syndrome presents as a spontaneous loss of flexor pollicis longus (FPL), FDP to the index and middle finger, and pronator quadratus muscle function with no associated sensory loss.^11^ When the electrodiagnostic studies and MRIs of patients with presumed isolated AIN palsies were reviewed, all patients in a recent series had MRI evidence of a more diffuse muscle involvement pattern without any radiologic signs of nerve compression of the AIN branch itself, which supports a broader inflammatory pathophysiology.^12^

Patients with recurrent CTS have improved symptoms for at least three months after surgery. Recurrence may be due to the development of perineural adhesions, fibrosis, or reconstitution of the transverse carpal ligament. Revision rates because of persistent or recurrent symptoms range from 1% to 5% in most large studies.^13^^,^^14^ Risk factors for revision surgery include older age, male sex, bilateral CTR, smoking, and rheumatoid arthritis.^13^ However, a meta-analysis of randomized controlled trials showed no differences in reoperation between open and endoscopic CTR.^15^ Finally, the development of new symptoms is most commonly the result of an iatrogenic injury to the median nerve, the palmar cutaneous branch of the median nerve, or the recurrent motor branch and requires operative repair or reconstruction.^14^ Prior to revision, a corticosteroid injection may be offered given that the sensitivity and positive predictive value for injection alone have been shown to predict the outcome of revision CTR in 87% of patients.^16^ Important diagnostic criteria to consider in the setting of recurrent CTS are detailed in Table 1.

**Table 1:** Diagnostic Criteria for Recurrent Carpal Tunnel Syndrome

Diagnosis of Recurrent Carpal Tunnel Syndrome	
History	Symptomatic improvement after initial release
	Nocturnal symptoms
Physical Examination	Positive Tinel sign over the carpal tunnel
	Positive Phalen test
	No sensory changes over PCBm distribution
	Negative Gainor compression test
Treatment	Symptomatic relief with CSI into the carpal tunnel
EMG	Confirm no intrinsic nerve pathology
	Document lack of nerve injury
Rule out “Mimickers”	TOS
	Cervical pathology
	Supracondylar process syndrome
	Pronator syndrome
	Lacertus syndrome
	AIN syndrome

CSI, corticosteroid injection; PCBm, palmar cutaneous branch of the median nerve.

### Revision surgeries

#### Repeat decompression and neurolysis

Both open and endoscopic releases are offered for primary decompression with similar outcomes. Although both approaches have acceptable reported outcomes in revision surgery, most prefer an open approach to fully and directly visualize the median nerve and surrounding structures. Repeat decompression involves extending the original incision proximally and distally to move outside the zone of potential scarring. The goal of external and internal neurolysis is to remove the perineural and intraneural fibrosis and improve nerve gliding. Neurolysis may be combined with tenosynovectomy, given that synovial hypertrophy is often seen during reoperation. In a recent meta-analysis by Soltani et al^17^ of surgical outcomes for persistent and recurrent CTS, all patients who underwent open decompression had external neurolysis but only four of the nine studies also performed internal neurolysis. The weighted success rate of these nine studies, which included 364 patients, was 75%.^17^ Similarly, in a meta-analysis of treatment outcomes for patients with recurrent CTS by Jansen et al,^4^ patients undergoing open decompression with or without neurolysis showed significant improvement as measured by the Boston Carpal Tunnel Questionnaire symptom severity and functional status scale.

#### Hypothenar fat pad flap

The vascularized hypothenar fat pad flap is a popular surgical technique for revision carpal tunnel surgery. Proponents of this technique assert that neurolysis alone is insufficient to prevent new scar formation. Therefore, the goal of the hypothenar fat pad flap and other pedicled or free flaps is to improve nerve coverage and neovascularization, thereby reducing fibrosis and constriction of the median nerve. The flap involves mobilizing a pedicled fat pad measuring approximately 4 cm x 3 cm from the hypothenar eminence and off the hypothenar musculature. The flap is interposed between the neurolyzed median nerve and the remaining radial leaf of the transverse carpal ligament with horizontal mattress sutures. A thin layer of adipose tissue should be left on the dermis when mobilizing the flap to allow for adequate vascularity. The flap is supplied by branches directly from the ulnar artery and branches of the ulnar artery to the small finger, the fourth webspace, and hypothenar muscles. In the meta-analysis by Jensen et al,^4^ there was a significant improvement in the Boston Carpal Tunnel Questionnaire symptom severity and functional status scale in patients receiving a hypothenar fat pad flap. Regarding the improvement size between the hypothenar fat pad flap group, the decompression-only group, the autologous fat transfer group, and the “other” group, there was a trend toward a greater improvement when using the hypothenar fat pad flap to treat recurrent CTS.^4^ Likewise, in the meta-analysis of Soltani et al,^17^ the flap group, which included the hypothenar fat pad flap and other pedicled and free flaps, demonstrated a success rate of 86%, significantly higher than the non-flap group. Factors associated with worse outcomes after a hypothenar fat pad flap include involvement of the nondominant hand, recurrence within a year from the previous surgery, scar tissue in the carpal tunnel, obesity, diabetes mellitus, and cervical spine problems.^18^

#### Other pedicled or free flaps

Several other local pedicled flaps can act as a barrier to prevent scarring around the median nerve. Proponents of the reverse radial artery fascial flap favor this option because of its significant size and bulk, long arc of rotation, and predictable vascular supply. In a series of six patients treated with a reverse radial artery fascial flap for recurrent CTS, all had good or excellent results.^17^ Similarly, in a series of 11 patients (13 hands) using the palmaris brevis turnover flap in recurrent CTS, subjective improvement ranged from 25% to 100%.^17^ The abductor digiti minimi, synovial, and radial/ulnar perforator flaps have also been used successfully in recalcitrant CTS. Several different free flaps, including scapular, lateral arm, and omental flaps, have been used to decrease scar adherence during revision surgery. Although most patients report improvement in symptoms following free flap surgery, many had donor site issues such as allodynia, itching, or the development of a ventral hernia.^4^ Thus, it is advisable to attempt a local, pedicled flap before considering free flap options. The authors would only consider a free flap in a rare scenario of having no local flap options available, such as in the setting of trauma. The various flap options are listed in Table 2.^19^, ^20^, ^21^, ^22^, ^23^, ^24^, ^25^, ^26^, ^27^, ^28^, ^29^

**Table 2:** Local and Free Flap Options for Recalcitrant Carpal Tunnel Syndrome

Flap Options for Revision Carpal Tunnel Surgery
Local Flaps
Hypothenar fat pad flap^19^
Reverse radial artery fascial flap^20^
Abductor digiti minimi flap^21^
Radial/ulnar perforator flap^22^^,^^23^
Synovial flap^24^
Palmaris brevis flap^25^
Pronator quadratus flap^26^
FDS flap^27^
Free Flaps
Scapular flap^28^
Lateral arm flap^28^
Omental flap^29^

#### Median nerve wraps

Another technique besides flaps used in recalcitrant CTS is wrapping the median nerve to insulate it and prevent the formation of new adhesions. Collagen matrix wraps and saphenous vein wraps are common choices that have been shown to treat 89% to 100% of patients successfully.^17^^,^^30^ In a series including six patients with recurrent CTS, saphenous vein wraps and neurolysis resulted in symptomatic improvement in all patients and improvement in sensory and motor nerve data in five of the patients.^31^ These wraps can be used in isolation or combination with other techniques. Spielman et al^32^ used a “triple-therapy approach” and treated 30 recalcitrant CTS patients with neurolysis, a collagen matrix collagen wrap, and a hypothenar fat pad flap with an 83% success rate. The authors suggested that although neurolysis is required to release the scar tissue, the collagen wrap is needed to act as a barrier to fibrous ingrowth. The hypothenar fat pad flap prevents reformation of the transverse carpal ligament and can help revascularize an ischemic median nerve.^32^ The authors used both NeuraWrap (Integra LifeSciences) and Axoguard Nerve Protector (AxoGen Inc) but did not compare the two wraps in the study. Although the NeuraWrap and Axoguard are now widely used in the setting of recurrent upper-extremity compressive neuropathies, no head-to-head studies to date directly compare their success.

### Author’s preference

**Figure 1:**
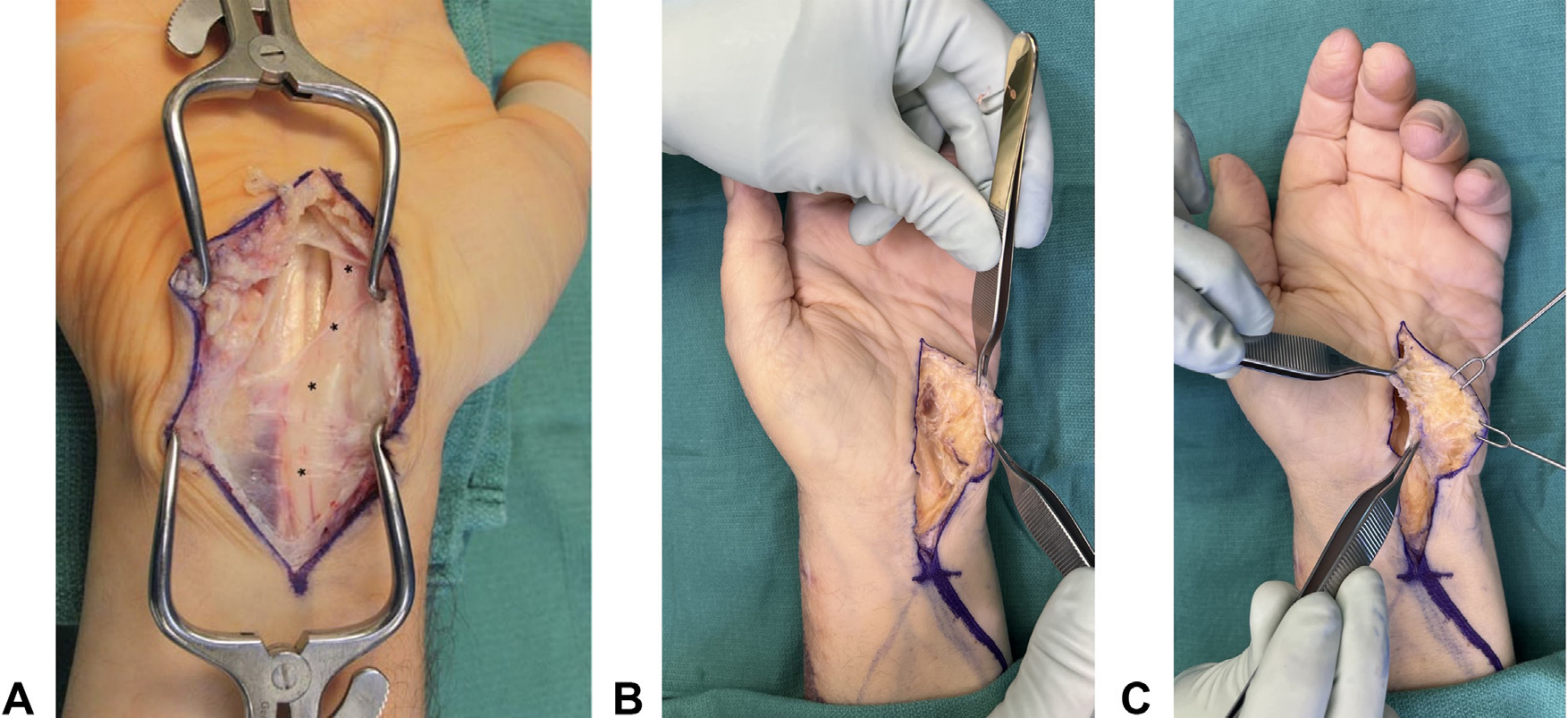
**A** Revision decompression of the median nerve (∗). **B** Elevation of a hypothenar fat pad flap for recalcitrant carpal tunnel syndrome. **C** Transposition of a hypothenar fat pad flap, so the median nerve is completely covered.

When treating patients with persistent CTS, we routinely examine them at monthly intervals to assess for symptom improvement. If patients have little or no improvement after three months or symptoms recur, we proceed with surgery. In addition, we would repeat an EMG at three months and reoperate if the findings are the same or worse compared with the preoperative study. The EMG is also useful in confirming no intrinsic nerve pathology and documents a lack of nerve injury. We would consider a diagnostic steroid injection prior to reoperation in equivocal cases. Physical examination findings consistent with persistent compressions, such as a positive Tinel sign at the wrist or a positive Durkan, Phalen, or carpal compression test, would push us to reoperate. Patients with an incomplete release after CTR may present with a magnification of their initial symptoms or significant discomfort. We would urgently take patients back to the operating room for exploration if they have worsening symptoms immediately after release, given that an iatrogenic nerve injury must also be ruled out with any worsening or new symptoms after CTR. For our revision carpal tunnel releases, we perform an extensile open CTR, ensuring that the transverse carpal ligament and antebrachial fascia are completely released. We also perform a hypothenar fat pad flap in all revision surgeries. The flap can be reliably mobilized and provides sufficient vascularized tissue to fully cover and insulate the median nerve, thereby preventing new adhesions and promoting nerve gliding. (Fig. 1 and Table 3). Patients are placed in a bulky, soft dressing for one week.

**Table 3:** Technical Considerations for the Hypothenar Fat Pad Flap

Technique for Hypothenar Fat Pad Flap
1. Perform after an extensile carpal tunnel release
2. Mobilize ulnarly based adipose flap of approximately 4 cm x 3 cm from the hypothenar eminence
3. Leave a thin layer of adipose tissue on the dermis to maintain adequate vascularity of the skin
4. Flap should provide complete coverage of the neurolyzed median nerve
5. Secure the flap to the radial leaf of the TCL using absorbable horizontal mattress sutures

TCL, transverse carpal ligament.

## Recalcitrant Cubital Tunnel Syndrome

### Anatomy and etiology

The ulnar nerve arises from the medial cord of the brachial plexus, which originates from the C8-T1 nerve roots. The cubital tunnel is the most common site of ulnar nerve compression. Many structures may be potentially compressing the nerve, including the Arcade of Struthers, the medial intermuscular septum, the medial epicondyle, Osborne’s ligament, and finally, under the flexor-pronator aponeurosis between the two heads of the flexor carpi ulnaris (FCU) muscle. The ulnar nerve then courses between the FCU and FDP muscles and enters the hand via Guyon canal, which is 40–45 mm in length and begins at the volar carpal ligament and ends at the fibrous arch of the hypothenar muscles. Within Guyon canal, the nerve branches into a superficial sensory and deep motor branch. It is important to be able to differentiate between cubital tunnel syndrome and the less common ulnar tunnel syndrome. Depending on the site of compression within Guyon canal, patients with ulnar tunnel syndrome may present with pain and paresthesias in the ulnar digits, weakness with pinch or grip, or a lack of fine motor control. On physical examination, patients may exhibit clawing of the ring and small fingers, atrophy of the hypothenar musculature, and have a positive Froment sign due to loss of thumb adduction. Unlike in cubital tunnel syndrome, sensation over the dorsal-ulnar hand should be intact since the dorsal cutaneous branch is proximal to Guyon canal. In terms of imaging, radiographs, including a carpal tunnel view, can identify a hook of hamate fracture but may be missed. Thus, a CT or MRI should be obtained if clinical suspicion is high. In addition, MRI can help to visualize a ganglion or ulnar artery abnormalities within Guyon canal. Other conditions that may present similar to cubital tunnel syndrome are cervical pathology, thoracic outlet syndrome (TOS), and brachial plexopathy. Therefore, a physical examination should include determining if there is cervical spine tenderness or a palpable mass, as well as assessing the supraclavicular region, shoulder girdle, and axilla for evidence of brachial plexopathy or TOS. Specific examination maneuvers that can help diagnose radiculopathy or myelopathy are the Spurling and Lhermitte maneuvers, while positive Wright or Adson maneuvers suggest TOS.^33^

Regarding carpal tunnel syndrome, it is critical to differentiate between persistent and recurrent symptoms. Incomplete release of all aforementioned compression sites, an anomalous anconeus epitrochlearis muscle, and a missed diagnosis of ulnar tunnel syndrome or cervical radiculopathy may all lead to persistent symptoms. The formation of perineural adhesions and fibrosis most commonly cause recurrent symptoms, whereas iatrogenic injury, ulnar nerve instability, and the formation of a medial antebrachial cutaneous nerve neuroma can present as new symptoms after the initial decompression. A summary of diagnostic considerations when addressing recurrent cubital tunnel syndrome is listed in Table 4. There are several methods of primary ulnar nerve decompression, including open versus endoscopic release, in situ release, subcutaneous transposition, and submuscular transposition. Overall, the incidence of persistent or recurrent cubital tunnel syndrome is approximately 25%, with no difference in failure rates between in situ release and anterior transposition.^34^ Younger age and a history of elbow trauma or cervical spinal disc herniation are associated with higher rates of revision surgery.^35^ When planning a revision procedure, it is important to ascertain the technique used in the primary decompression. Nerve conduction studies, EMG, and ultrasound may also be helpful adjuncts before surgery in determining the severity of the disease, degree of perineural scarring, the dynamic position of the nerve, and the presence of neuromas. In addition, it is important to set expectations with patients before surgery that the results from revision surgery are often worse than primary surgery, and complete symptom resolution is unlikely. A case-control study showed that both subjective symptoms, as measured by the Levine-Katz questionnaire and the Patient-Rated Elbow Evaluation, and objective measurements of two-point discrimination and pinch strength are worse in the revision group than in the primary decompression group.^36^ Similarly, 21% to 25% of patients undergoing revision surgery had either no change or a worsened McGowan grade after surgery.^36^^,^^37^

**Table 4:** Diagnostic Criteria for Recurrent Cubital Tunnel Syndrome

Diagnosis of Recurrent Cubital Tunnel Syndrome	
History	Period of symptomatic improvement after initial release
Physical Examination	Positive Tinel sign over the cubital tunnel
	Symptomatically unstable ulnar nerve
	Negative Spurling maneuver
Imaging	CT or MRI if concern for the hook of hamate fracture
	MRI if suspicious for ganglion or ulnar artery abnormalities within Guyon canal
EMG	Same or worse findings at six months after surgery compared to preoperative findings
Rule out “Mimickers”	TOS
	Cervical pathology
	Brachial plexopathy
	Ulnar tunnel syndrome

### Revision surgeries

#### Revision neurolysis

Performing external neurolysis alone is relatively uncommon in revision cubital tunnel surgery. Many fear that the adhesions will redevelop after a revision surgery that does not provide any barrier to the nerve. Thus, external neurolysis is often performed concurrently with fat flaps or nerve wrapping. Nevertheless, eight patients reported fair or good outcomes in a series of nine patients treated with external neurolysis alone after failed anterior submuscular transposition.^38^ Yushan et al^39^ also demonstrated a 95% patient satisfaction rate after 21 patients were treated with neurolysis and ulnar groove plasty.

#### Submuscular transposition

Submuscular transposition has been considered by many to be the preferred treatment for recalcitrant cubital tunnel syndrome and remains the most common type of revision surgery.^38^ The technique involves releasing the flexor-pronator mass, transposing the nerve under the muscle to provide a well-vascularized course, and subsequent muscle repair.^40^ A literature review by Wever et al^41^ reported that the clinical success rate after submuscular transposition ranges from 25% to 100%, with a mean subjective improvement rate of 63%. Musculofascial lengthening can be performed with submuscular transposition in order to increase the submuscular space for the nerve and prevent subsequent compression by the flexor-pronator mass.^41^

#### Subcutaneous transposition

Many studies have challenged the notion that submuscular transposition is the optimal revision surgery. Caputo et al examined 20 revision patients who underwent subcutaneous transposition regardless of the primary surgery and showed that 15 had good or excellent results.^42^ Given that the subcutaneous transposition is at least as effective as the submuscular transposition and results in less morbidity, it should be considered a reliable option. While the nerve is often secured anteriorly with a fascial flap, a pedicled adipofascial flap based on perforators from the brachial artery is becoming an appealing alternative because it can reduce nerve adherence and scarring and provide an optimal environment for vascular regeneration, similar to the concept behind the hypothenar fat pad flap. The adipofascial flap has been successfully used in primary cubital tunnel release with subcutaneous transposition with equal to superior results compared to a fascial sling.^43^ Pagnotta et al^44^ recently used the adipofascial flap without transposition in a series of eight patients with recalcitrant cubital tunnel syndrome. All patients had good 4-year outcomes with a return to daily activity and work.^44^

#### Medial epicondylectomy

Medial epicondylectomy is more commonly performed in primary cubital tunnel surgery, although Gaspar et al^45^ reported that it is associated with a 13% reoperation rate. The authors find that the risk of revision surgery is higher in younger patients who have less severe disease, are taking opioids before surgery, and have associated workers' compensation claims.^45^ Papatheodorou et al^38^performed a minimal medial epicondylectomy, if not previously performed, on all of their revision cases, in addition to applying a porcine extracellular matrix wrap with an overall improvement in patient satisfaction, grip strength, and pinch strength. The medial epicondylectomy is meant to prevent tethering and allow for better excursion of the ulnar nerve.^38^

#### Nerve wrapping

**Figure 2:**
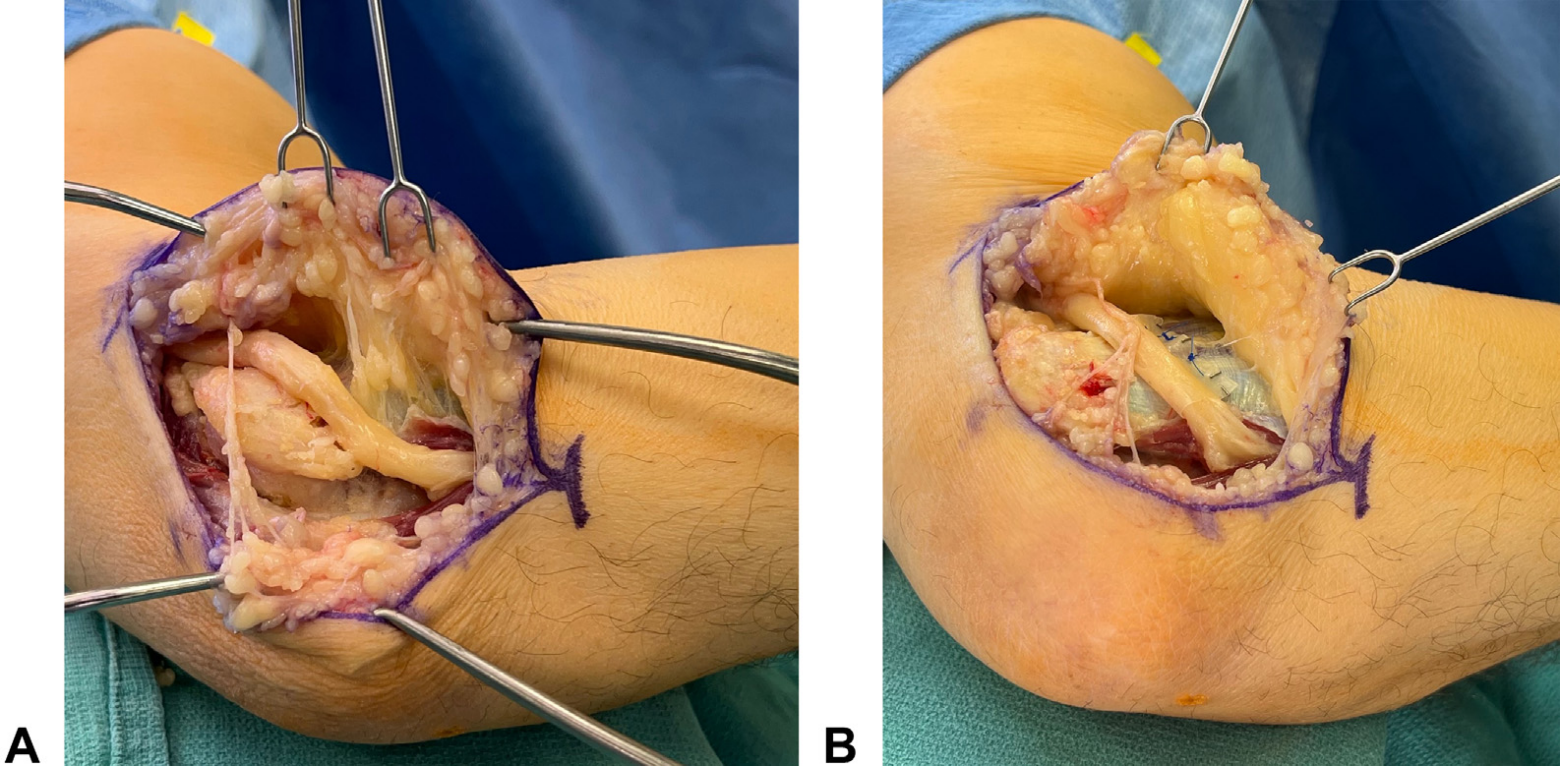
**A** Anterior subcutaneous transposition of the ulnar nerve after decompression. **B** A 10 mm x 40 mm Axogen nerve protector is loosely wrapped around the nerve at the level of the medial epicondyle. The nerve wrap is then sutured to the flexor-pronator mass, and stable anterior transposition is ensured through a full range of motion.

Wrapping the ulnar nerve with autogenous, allogenic, or xenographic materials in the setting of revision cubital tunnel surgery can help prevent recurrent adhesions. Figure 2 shows the anterior transposition of the ulnar nerve secured with an Axogen nerve protector, which is made of porcine small intestine submucosa. Amniotic membrane has been used in a series of eight patients with 100% satisfaction and significant improvements in the DASH (Disabilities of the Arm, Shoulder, and Hand) scores.^46^ Similarly, 83% of patients reported improvement after revision using a collagen nerve wrap.^19^ The saphenous vein can act as an adhesion barrier and was 100% effective in improving pain in a series of 17 patients undergoing revision surgery.^38^ The vein is easily harvested with a vein stripper and split longitudinally to form a long rectangle. The intima of the vein is placed against the nerve and wrapped circumferentially. Finally, the porcine extracellular matrix has successfully been used in revision operations with improved pain, grip strength, and two-point discrimination.^38^

### Author’s preference

**Figure 3:**
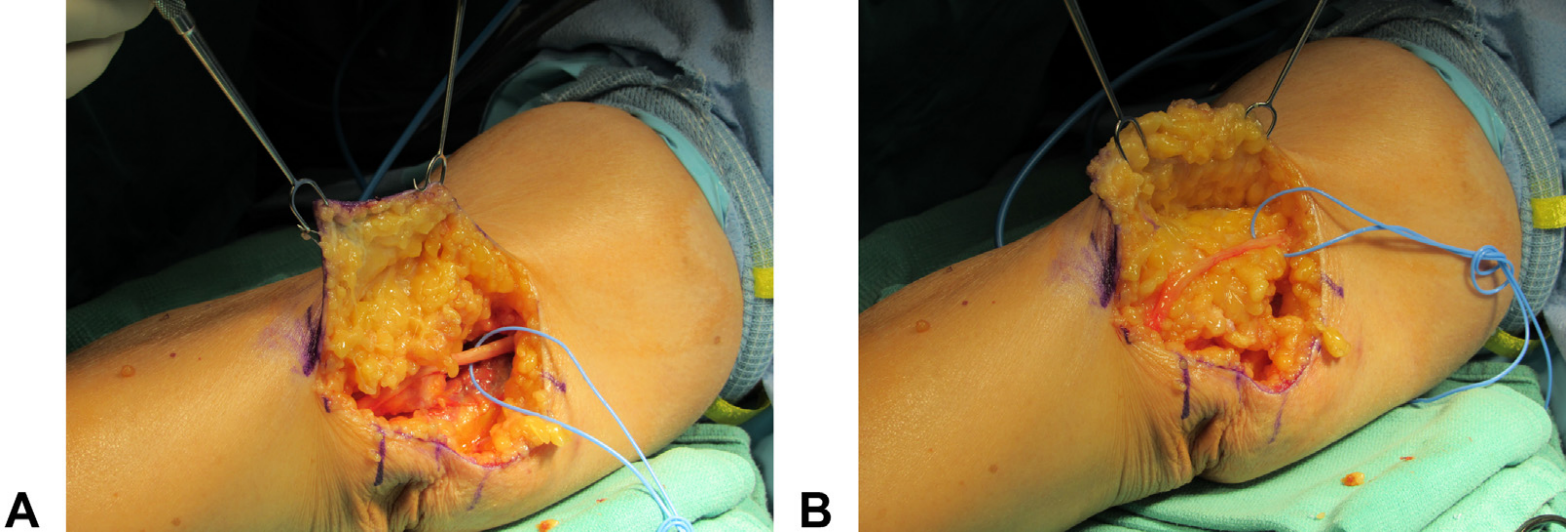
**A** Elevation of an adipofascial flap for recalcitrant cubital tunnel syndrome. Courtesy of Dr Marc Richard. **B** Inset of an adipofascial flap posterior to the ulnar nerve. Courtesy of Dr Marc Richard.

For most primary cubital tunnel releases, we perform an open in situ decompression and ensure all apparent and potential compression sites are adequately released, including snapping triceps, Osborne’s ligament, and the aponeurosis of the two heads of FCU.^47^ Patients may take slightly longer to recover after primary decompression, so we do not entertain revision until symptoms have persisted for at least six months or if symptoms recur after a period of relief. As with revision CTS, we would reoperate if patients have worsening symptoms immediately after release or if physical examination findings are consistent with persistent compressions, such as a positive Tinel sign at the cubital tunnel. Similarly, we would refuse a reoperation if an EMG at six months demonstrated the same or worse findings than the preoperative study. Finally, we would reoperate for an ulnar nerve that is symptomatically unstable. For revision surgery, we perform neurolysis, anterior subcutaneous transposition, and an adipofascial flap, as described by Rosenwasser^43^ (Fig. 3). Technical considerations for this flap are listed in Table 5. This technique is also used in primary procedures with symptomatic or significant ulnar nerve instability. We believe submuscular transposition increases surgical morbidity, and there is no convincing evidence of superior outcomes. After surgery, patients are placed in a soft dressing and encouraged to begin a gentle range of motion early after surgery.

**Table 5:** Technical Considerations for the Adipofascial Flap

Technique for Adipofascial Flap
1. Perform after ulnar nerve decompression and anterior subcutaneous transposition
2. Mobilize the adipose flap of approximately 12 cm x 4 cm from the medial elbow
3. Leave a thin layer of adipose tissue on the dermis to maintain adequate vascularity of the skin
4. Take care to avoid injury to the adjacent branches of the medial antebrachial cutaneous nerve
5. Transpose the flap, so it encircles the nerve in a posterior-to-anterior direction
6. Suture the flap to itself with absorbable sutures
7. Range elbow to ensure free nerve gliding and no kinks

## Conclusions

Revision CTR and cubital tunnel surgery can improve upper-extremity pain and strength in properly selected patients. The outcomes of the various techniques are difficult to compare owing to the lack of standardized and quantitative reporting and randomized controlled trials. For recalcitrant CTS, we advocate an open approach with a hypothenar fat pad flap. For recalcitrant cubital tunnel surgery, we perform repeat neurolysis, subcutaneous transposition, and an adipofascial flap. In revision procedures, covering the nerve with vascularized tissue helps to prevent perineural adhesions and promotes neovascularization. In conjunction with thorough decompression, this facilitates the recovery of the nerve.
